# Effects of *Yersinia ruckeri* invasion on the proteome of the Chinook salmon cell line CHSE-214

**DOI:** 10.1038/s41598-020-68903-5

**Published:** 2020-07-16

**Authors:** Simon Menanteau-Ledouble, Katharina Nöbauer, Ebrahim Razzazi-Fazeli, Mansour El-Matbouli

**Affiliations:** 1 Clinical Division of Fish Medicine, University of Veterinary Medicine, Veterinärplatz 1, 1210 Vienna, Austria; 2 VetCore Facility for Research, University of Veterinary Medicine, Vienna, Austria

**Keywords:** Applied microbiology, Cellular microbiology, Pathogens

## Abstract

*Yersinia ruckeri* is an important bacterial pathogen of fish, in particular salmonids, it has been associated with systemic infections worldwide and, like many enteric bacteria, it is a facultative intracellular pathogen. However, the effect of *Y. ruckeri*’s interactions with the host at the cellular level have received little investigation. In the present study, a culture of Chinook Salmon Embryo (CHSE) cell line was exposed to *Y. ruckeri*. Afterwards, the proteins were investigated and identified by mass spectrometry and compared to the content of unexposed cultures. The results of this comparison showed that 4.7% of the identified proteins were found at significantly altered concentrations following infection. Interestingly, infection with *Y. ruckeri* was associated with significant changes in the concentration of surface adhesion proteins, including a significantly decreased presence of β-integrins. These surface adhesion molecules are known to be the target for several adhesion molecules of *Yersiniaceae*. The concentration of several anti-apoptotic regulators (HSP90 and two DNAj molecules) appeared similarly downregulated. Taken together, these findings suggest that *Y. ruckeri* affects the proteome of infected cells in a notable manner and our results shed some light on the interaction between this important bacterial pathogen and its host.

## Introduction

*Yersinia ruckeri* is an important fish pathogen, mostly known as the causative agent of septicaemic infections in salmonids, sometime referred to as “enteric redmouth disease”, although it is able to infect a large number of fish species^1,2^. *Y. ruckeri* belongs to the *Yersiniaceae* family and like many members of the *Enterobacteriaceae* and *Yersiniaceae* families, it is a facultative intracellular pathogen^3,4^, likely existing within cytoplasmic vacuoles^5^, although the subject has received little discussion in the literature. Intracellular location is beneficial for bacteria as it allow them to access nutrients that are sequestered away by the host organism as well as shield the bacterium from the immune system and several therapeutants. Moreover, it might constitute a mean for the bacteria to cross biological membranes and gain access to the circulatory system.

Notably, several bacterial pathogens are known to alter the gene expression of their hosts. For example, the type 3 secretion system (T3SS) is a virulence factor that specialises in interfering with host cell processes through the injection of effector proteins directly into the cytoplasm of the host cells^6^. The T3SS of enteropathogenic and enterohaemorrhagic *Escherichia coli* (EPEC and EHEC) translocates the intimin receptor (Tir) into the host’s enterocytes to act as a target for the bacterium’s adhesion molecules^7,8^. Similarly, multiple T3SS exist in *Yersiniaceae*, including Ysa which is similar to the T3SS carried on the *Salmonella* pathogeny island^9^ and which has been associated with the survival of the bacterium intracellularly in drosophila cells^10^. Interestingly, *Y. ruckeri* has been shown to possess a Ysa T3SS^11^ and analysis of the bacterium’s genome has allowed to identify several more genes that appear to be part of this T3SS, including *sptp* (NJ56_03895) that is predicted to encode an effector protein^12^. However, virtually nothing is known about the T3SS of *Y. ruckeri*.

Moreover, even cells that do not belong to the myeloid lineage still react to the presence of microbial associated molecular patterns (MAMPs). For example, most eukaryotic epithelial cells are known to express Toll-like receptors (TLR)^13,14^. Activation of TLR is followed by the initiation of the nucleotide-binding oligomerization domain (NOD) and signalling cascades that alter the expression of multiple genes within the cells and result in the expression of signalling molecules such as interferons as well as cytokines and chemokines^14^. Salmonid fish are also known to express TLRs, including several unique to teleosts, however the complete repertoire of their substrates is still uncatalogued and the specific molecular patterns recognise by several salmonid TLRs remain to be described^15^.

However, while some studies have previously reported on the effect of *Y. ruckeri* infection on the proteome of fish organs, these were conducted at the fish levels and none has focussed on these interactions on individual cells. Consequently, in the present manuscript, we report on the effects of *Y. ruckeri* interactions on the protein expression in a fish cell line.

## Results

A total of 1614 different salmonid proteins were used for quantification (see Supplementary Table 1). Of these proteins, 76 (4.7%) were found to be significantly differentially expressed in infected cells compared to the control. These differentially expressed proteins were almost evenly distributed with 36 proteins (47.3% of the differentially expressed proteins) present at higher levels and 40 proteins (52.6%) present at a significantly lowered level. Moreover, the 20 proteins that were found at the most highly increased concentrations and the 20 proteins found at the most highly reduced concentrations in the infected samples were identified and analysed using Uniprot (see Supplementary Table 2). In addition, their protein–protein interactions visualised based on the Search Tool for the Retrieval of Interacting Genes/Proteins (STRING; STRING consortium).

**Figure 1:**
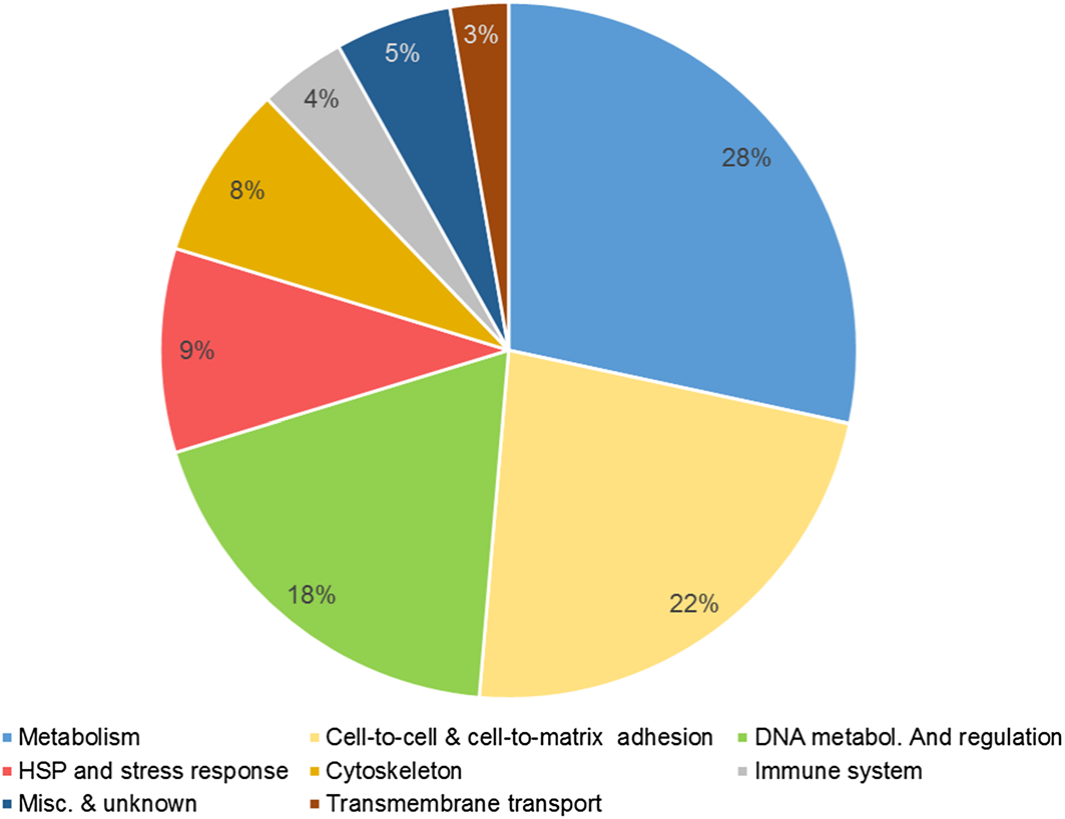
Relative distribution of the differentially expressed genes, classified per function.

Most of the proteins found at significantly altered levels belonged to one of three categories (Fig. 1 and Tables 1 and 2): protein involved with the cell metabolism (28%), protein involved in cellular adhesion to either the cellular matrix or other cells (22%) and proteins involved in the DNA metabolism and regulation of gene expression (18%). Repartition of the proteins functions was different between the proteins found at a higher and lower level, however, with almost half (41.7%) of the proteins found at a higher level being involved in the cellular metabolism. Notable other functions were cell-to-cell adhesion (19.4%) and DNA metabolism and regulation (13.9%). In contrast, the proteins most commonly found at lower-levels were involved in cell-to-cell-adhesion (25.0%) and DNA metabolism and regulation (22.5%) with only 15.0% of these proteins being involved in cell metabolism.

**Table 1:** Repartition of the function of the differentially expressed proteins in Chinook salmon cell line CHSE-214 infected with *Yersinia ruckeri* ATCC 29473.

Function	Proteins found at higher levels	Proteins found at lower levels	All differentially expressed proteins
Metabolism	15 (42%)	6 (15%)	21 (28%)
Cell-to-cell and cell-to-matrix adhesion	7 (19%)	10 (25%)	17 (22%)
DNA metabol. and regulation	5 (14%)	9 (22%)	14 (18%)
HSP and stress response	2 (6%)	5 (12%)	7 (9%)
Cytoskeleton	1 (3%)	5 (12%)	6 (8%)
Misc. and unknown	1 (3%)	3 (7%)	4 (5%)
Immune system	3 (8%)	0 (0.0%)	3 (4%)
Transmembrane transport	1 (3%)	1 (3%)	2 (3%)
Apoptosis	1 (3%)	1 (3%)	2 (3%)
Total	36	40	76

**Table 2:** Most differentially expressed proteins in the study.

Accession number	Protein name	log2_FC
**2a—most highly up-regulated protein**		
XP_013982441.1	Transcriptional repressor CTCF-like isoform X1	**20.11702**
XP_014004191.1	Fibrillin-2-like	**19.44886**
XP_014022394.1	Microfibrillar-associated protein 2 isoform X1	**6.209489**
XP_013992610.1	Creatine kinase M-type-like	**4.29486**
XP_014060037.1	Serine protease HTRA1A-like	**2.981173**
XP_014011095.1	Metastasis-associated protein MTA2-like isoform X1	**2.601636**
XP_014041586.1	Thrombospondin type-1 domain-containing protein 4-like	**2.537307**
XP_014005671.1	A disintegrin and metalloproteinase with thrombospondin motifs 5-like	**2.526878**
XP_014018631.1	Dehydrogenase/reductase SDR family member 13-like isoform X1	**2.475252**
XP_014023923.1	Constitutive coactivator of PPAR-gamma-like protein 1 homolog isoform X1	**2.340926**
XP_014017796.1	Histone H3.3	**2.276828**
XP_014046901.1	Histone H4	**2.233742**
XP_014070509.1	Zinc finger protein 883-like	**2.049265**
XP_013981166.1	Probable inactive glycosyltransferase 25 family member 3	**2.004491**
XP_014036170.1	Sickle tail protein homolog isoform X1	**1.996413**
XP_014065419.1	Structural maintenance of chromosomes protein 2, partial	**1.943045**
XP_013999830.1	Band 4.1-like protein 2 isoform X1	**1.910006**
XP_014034449.1	ATP-citrate synthase-like isoform X1	**1.899294**
XP_014028138.1	Acetoacetyl-CoA synthetase	**1.843624**
XP_014043980.1	Collagen alpha-1(VI) chain-like	**1.831625**
**2b—most highly down-regulated protein**		
XP_014014203.1	Polymerase I and transcript release factor	− **19.6531**
XP_014029288.1	Integrin alpha-4	− **19.3051**
NP_001133388.1	Filamin-binding LIM protein 1	− **18.7672**
XP_013993457.1	Drebrin-like isoform X1	− **7.88954**
NP_001133495.1	Ribonucleoside-diphosphate reductase subunit M2	− **3.34038**
XP_014025930.1	DnaJ homolog subfamily A member 4-like	− **2.92876**
XP_014021642.1	Ehrin type-A receptor 2 isoform X1	− **2.84179**
XP_014016754.1	S-adenosylmethionine synthase isoform type-2-like	− **2.81203**
XP_014033914.1	Uncharacterized protein LOC106588899	− **2.45663**
XP_013986821.1	Importin subunit alpha-1-like	− **2.42589**
XP_014022166.1	Four and a half LIM domains protein 3-like isoform X1	− **2.32055**
XP_013988049.1	Erythrocyte band 7 integral membrane protein-like isoform X1	− **2.29836**
XP_014046665.1	Calponin-2-like	− **2.21518**
XP_014034982.1	Integrin beta-4 isoform X1	− **2.16874**
XP_013987582.1	Intermediate filament protein ON3-like	− **2.16488**
XP_014013582.1	Integrin beta-4-like	− **2.14322**
XP_014018714.1	Serpin H1-like	− **2.0325**
XP_014019725.1	WD repeat-containing protein 75 isoform X1	− **2.0256**
XP_013982302.1	Sorbitol dehydrogenase-like isoform X1	− **2.01695**
XP_014068817.1	ES1 protein homolog, mitochondrial-like	− **1.95292**

Bold values indicate the two sub-categories: proteins found at higher concentrations and proteins found at lowered concentrations.

Bacterial invasion appeared to induce the expression of two proteins that were not found in the non-infected samples. The first of these was the transcriptional repressor CTCF-like and the other was the fibrillin-2 protein, which is a protein involved in the cellular attachment to connective tissue.

Conversely, several proteins appeared absent from the infected samples, suggesting that their expression had been turned off or reduced below the detection limit following the infection. These included the polymerase I and transcript release factor also known as the caveolae associated protein 1 (Cavin1). Similarly, the extracellular adhesion matrix proteins integrin alpha-4, and filamin-binding IM protein 1 could not be detected in the infected cells.

Moreover, several other proteins that were found in the uninfected samples were found at significantly higher levels in the infected ones. This included another protein involved in the cell’s attachment: microfibrillar-associated protein 2. Other proteins that were strongly differentially expressed included the creatine kinase M-type like as well as the serine protease HTRA1A, an enzyme involved in a variety of functions and with a wide arrays of target, including extracellular matrix proteins and proteoglycans.

Among the proteins that appeared under-expressed following bacterial infection were two isoforms of integrin-β: the integrin beta-4 isoform X1as well as the integrin beta-4 like. Similarly, a homolog of the collagen chaperon Serpin H1 was also present at lower levels in the infected cells.

Other proteins that were present at lower levels included ephrin type A receptor (EphA1) which is involved in cell to cell signalling, including expression of integrin molecules as well as a homolog of calponin 2 which is involved in the cytoskeleton apparatus and cell migration. Several regulators were also present at lower levels including both DNAj (also known as HSP40) homolog subfamily A and subfamily B as well as the heat shock protein HSP90. Notably, the abundance of the interferon regulatory factor 2-binding protein 2-B-like, an important marker of the cellular response to infection, was only found to be slightly altered, suggesting that it was only slightly under-expressed.

Analysis of protein–protein interactions showed that all but one of the most highly differentially regulated proteins were part of a network or interacting protein (see Supplementary Table 2).

## Discussion

Only one time point was considered in this experiment and no cytopathic effect (CPE) was observable at this time point. This sampling time was based on our previous work, which indicated that cytopathic effects developed after this time point with the host cells dying following a few hours of infection. Within 6 h of infection, a large portion of the cell monolayer was destroyed and CPE were clearly visible with the naked eyes^3^. To avoid the confounding effects that cell death would bring on the cells’ proteome, it was elected not to investigate later time points.

Exposure to *Y. ruckeri* significantly altered the presence of 76 proteins, representing approximately 4.7% out of all 1614 of the host proteins quantified. A significant number of the differentially expressed proteins were involved in cellular adhesion and adhesion to the extracellular matrix. This is consistent with the fact that multiple cell to cell and cell to matrix adhesion molecules have been identified as target for *Y. ruckeri* in the past^16,17^. Interestingly, more proteins involved in the cellular metabolism appeared to be up-regulated than down-regulated. This might be indicative of the effort and energy expenditure in response to the infection, for example to power the rearrangement of the cytoskeleton that is associated with such infections.

Among the notable proteins whose expression was triggered in infected cells was the CCCTC-binding factor (CTCF), a regulatory protein involved in a wide-array of functions^18^. In particular, it has recently been shown that CTCF plays a role in homogenising gene expression in cells^19^. These findings could be explained by the fact that CTCF has been shown to be up-regulated by NF-κB^20^, as it is well established that NF-κB is a marker of inflammation and is stimulated in the presence of MAMPs. For example, it has recently been shown that presence of flagellin molecules from *Y. ruckeri* induced expression of NF-κB both in vivo and in vitro in channel catfish (*Ictalurus punctatus*)^21^. In mammals, infection of the epithelial cell line HT-29 and T84 with *Salmonella* was similarly followed by an over expression of NF-κB^22^. Similarly, infection of human bronchial epithelial cells (BEAS-2B) by *Bordetella pertussis* resulted in an over-expression of genes regulated by NF-κB^23^.

The association of creatine kinase M-type with *Yersinia* infection is not clear. This enzyme plays an important role in ATP metabolism, especially in the muscles and its increased level in the blood stream is generally regarded as a marker of tissue damage, for example during bacterial infections^24^, including with *Yersinia enterocolitica*^25^. Increased production in cultures of epithelial cell has, to the best of our knowledge, not been reported but might plausibly be a response to the energy burden of the infectious process. The cytoskeletal rearrangement, for example, is dependent on the availability of ATP^26^.

On the other hand, other molecules were not detected in infected cells in contrast to non-infected cells. These included the actin-binding cytoskeletal protein calponin 2. Calponin 2 is a known substrate for Protein Kinase C. Furthermore, calponin 2 is involved in the phosphorylation of protein kinase C and it has been suggested that a major role is to connect the Protein Kinase C cascade to the mitogen-activated protein kinase pathway^27,28^. Interestingly, it has recently been demonstrated that the cytotoxic necrotizing factor toxin (CNF) of *Y. pseudotuberculosis* had a negative effect on the presence of calponin 2^29^.

Similarly, Cavin1 appeared to be silenced following infection. Cavin1 is involved in the formation of caveolae, membrane structures involved in the transport of compounds across the membrane, including the formation of endocytic compartments^30^. Caveolae can be co-opted for bacterial invasion^31^ and have been theorised to play a role in the uptake of the type 3 effector protein YopM of *Y. pestis*^32^. Interestingly, caveolae proteins are known to be activated by protein kinase C, therefore it is not unexpected for the abundance of Cavin1 to parallel that of Calponin 2^33^. Changes in Cavin1 expression are likely related to disturbances of the cytoskeletal apparatus.

Taken together, the expression of extracellular adhesion molecules showed contradictory patterns: Expression was greatly increased for the fibrillin-2 protein and microfibrillar-associated protein 2 as well as the serine protease HTRA1A. These proteins play a role in various functions including attachment to the extracellular matrix proteins and regulation of growth factors^34–36^. Conversely, other extracellular adhesion proteins such as and filamin-binding IM protein 1 and the integrin alpha 4 as well as two isoforms of integrin-β (integrin β-4 and integrin β-4 like) were found at significantly decreased levels in infected cells. This is particularly relevant because transmembrane cell-adhesion proteins such as integrins are known as the targets for the attachment of *Yersiniaceae*. More particularly, it has been shown in the human pathogens *Y. enterocolitica* and *Yersinia pseudotuberculosis* that two adhesin molecules, invasin and YadA are able to bind on the β1-integrins^37–39^. Such adhesion initiate a tight junction and is followed by receptor clustering and the phosphorylation of tyrosine kinase FAK leading to cytoskeletal rearrangement and the ingestion of the bacterium^40–42^. This mechanism of intracellular invasion has been described as the “zipper mechanism”^40^ and is considered a way for bacterial pathogens to cross epithelial membranes and gain entrance into the host^39,43^. The mechanisms of invasion and intracellular entry of *Y. ruckeri* have not yet received the same amount of scrutiny. However, like *Y. enterocolitica* and *Y. pseudotuberculosis*, it is a known facultative intracellular pathogen^3, 4^. Recently, it was shown that silencing of the integrin β-1 precursor in an in-vitro culture of Chinook Salmon embryo cell culture (CHSE) resulted in a significant reduction of the cells’ suceptibility to invasion by *Y. ruckeri*^44^ and it is therefore very plausible that *Y. ruckeri* can make use of the zipper mechanism to invade host cells. In this context, the fact that several adhesion molecules, and in particular integrin molecules were detected at a lower level was unexpected. This might be related to the findings that the regulator EphA1 was also under-expressed as this molecule is involved in the regulation of several other proteins, including integrin activity and can promote cell adhesion^45^.

Furthermore, both HSP90 and homologs DNAj subfamily A and DNAj subfamily B were found to be present at much lower numbers in the infected cells. All of these molecules have been reported to be involved in the regulation of apoptosis and have anti-apoptotic properties^46–48^. Because apoptosis and pyrotosis are mechanisms through which organisms limit the spread of pathogen, especially intracellular pathogens^49,50^, including *Yersiniaceae*^51^, the decreased expression of these regulators is consistent with the cells promoting programmed cell death in response to bacterial invasion.

Also noteworthy was the absence of immune proteins. This absence included tumour necrosis factors as well as interleukin or major histocompatibility complexes (the minor histocompatibility antigen H13-like was however present at significantly higher levels). Similarly, the interferon regulatory factor 2-binding protein 2-B-like was not found to be significantly differentially expressed (*p* = 0.535), which suggests that the immune response of the cells was limited. *Y. ruckeri* is known to have immunosuppressive properties, notably; it was recently reported that this bacterium can suppress the myeloid differentiation factor 88 (MyD88)^52^. Because MyD88 has been shown to be required for interferon signalling following bacterial infection in *Brucella abortus*, this would be consistent with the present findings^53^. Notably, Myd88 independents activation has also been described for NF-κB and some interferons^54^, so this picture is likely complex.

When compared to other studies of the response of epithelial cells to bacterial infection, our results appear quite different. Eckmann et al*.* reported that only a small fraction of the genes showed significantly different expression levels following infection with *Salmonella*, with expression ratios of 2 or higher (about 20 out of 4,300 genes investigated)^22^. On the other hand, we found 76 out of 1,614 with differential expression levels above that threshold. Moreover, these authors found that many signalling molecules were differentially expressed and none of the surface adhesion molecules while our results show the opposite^22^. There could be several explanation for these differences, for example, this could be due to differences in the two bacterial species. Interestingly, while a member of the *Yersiniaceae* family, the virulence of *Y. ruckeri* present multiple similarities with that of *Salmonella*. For example, the T3SS of *Y. ruckeri* is in fact most closely related to the T3SS carried on *Salmonella* pathogenicity island 1 (SPI-1) of *Salmonella enterica*^12,55^. Another plausible explanation would be differences in cell lines used. For example, both HT-29 and T84 are derived from cancerous cells while CHSE are embryo derived and this likely alters the expression of several genes.

Similarly, one of the findings recently reported by Wang et al*.*^56^ was that epithelial cells harvested from the intestine of rainbow trout and exposed to lipopolysaccharide (LPS) did express multiple inflammatory cytokines, including interleukin and tumour necrosis factors. In addition, the E-cadherin attachment protein was found to be differentially expressed, however, E-cadherin was not found to be present at significantly different level in our experiment (*p* = 0.433). On the other hand, the attachment proteins that were found to be differentially expressed in our experiment were not commented upon by Wang et al*.* Similarly, these authors found the F-actin protein to be significantly over expressed following exposure to LPS, while in our experiment, the F-actin was not observed to be present at significantly different levels (*p* = 0.855) and HSP-70 that was found to be differentially expressed by Wang et al. was not detected in our experiment^56^. The reason for these differences is difficult to ascertain. It might be due to the difference in cell types or to the fact that Wang et al. used pure LPS while we exposed the cell to live bacteria: bacteria harbour many more MAMPs beside LPS, each of which might have its own effect; moreover, they are known to actively steer and subvert the cellular response^57^.

It is likely that the discrepancies observed are a combination of different factors: *Y. ruckeri* is considered an atypical member of the *Yersiniaceae* family and so, it is conceivable that its effect on invaded cells would be somewhat different. Furthermore, most of the studies quoted were conducted on mammalian cells or freshly harvested cells. In the future, it might be of interest to investigate the effect of inactivated bacteria as well as the response of other cell types to these bacteria to better differentiate what part of the changes is caused by the response of the cells to the bacterial presence and what is part is caused by the bacteria’s interference.

## Conclusion

In the present study, we investigated the effects of *Y. ruckeri* infection on the proteome of a monolayer of the epithelial cell line CHSE. Infection had a significant effect on the abundance of a large number of the identified proteins. Interestingly, the most significant changes were in the levels of several surface adhesion molecules as well as in regulatory protein controlling their expression, for example, two isoforms of integrin-β: integrin beta-4 isoform X1 and integrin beta-4 like were found to be significantly under-represented in the infected samples. Intriguingly, these findings were quite different from the ones reported by previous authors using different cell type and bacteria or MAMPs. Taken together, these findings illustrate the extent of the changes caused by bacterial infection and the ability of bacterial pathogen to alter the proteome of infected cells.

## Methods

### Bacterial isolates and cell strains

The bacterium used in this experiment was *Y. ruckeri* ATCC 29,473, a type strain that was initially isolated from a clinical case in Rainbow trout (*Oncorhynchus mykiss*). The bacterial isolate was stored at − 80 °C until two days before the experiment.

The cell line used was Chinook salmon embryonic cells (CHSE-214) that was initially isolated from *Oncorhynchus tshawytscha* in 1964^58^. It has been reported that some lines of this cell line were in fact contaminated with cells from Bluegill (*Lepomis macrochirus*). However, a molecular investigation conducted in our laboratory using five primer pairs designed to be specific for *O. tshawytscha* as well as five primer pairs specific for *L. macrochirus* allowed to confirm that the lines present in our clinic did in fact originate from *O. tshawytscha*. This cell line was maintained in Minimum Essential Medium Eagle (MEM; Gibco).

Both the bacterial isolates and the cell line were elected based on our previous experience that had determined that they were both suitable, in particular for intracellular invasion and allowed us to optimise the infection protocol and sampling times for this experiment^3^.

### Bacterial infection procedure

Two days before the infection procedure, *Y. ruckeri* ATCC 29,473 was recovered from the freezer, inoculated on LB agar, and incubated at 22 °C. After 36 h, a single colony was picked up from the plate and used to inoculate 7 ml of LB broth. The broth was incubated overnight at 25 °C with shaking at 225 RPM. At the end of this incubation period, the optical density of the bacterium was measured using a spectrophotometer at 600 nm (Eppendorf Biophotometer) and adjusted to 0.5. Then, 5 ml of the culture were pelleted by centrifugation at 5,000g at room temperature (cooling centrifuge 5,810 R, Eppendorf) for five minutes before being resuspended in 50 ml of MEM.

In the meantime, CHSE cells were cultivated in 25 ml flasks in seven replicates. The cells were observed using an inverted microscope to confirm that the cultures were healthy and near confluent. Then, in three randomly selected flasks, the culture medium was removed and replaced with the bacteria inoculated MEM while in three other flasks, the medium was replaced with fresh, sterile MEM. The bacteria were left to interact with the cells for 2 h, a period of time elected based on our previous experiment that allowed for the bacteria to invade the cells and affect their expressome while still being too short to cause death and lysis in the infected cells^3^. Afterwards, the cells were lysed and the proteins were harvested as described below.

In the last remaining flask, the cells were harvested using a cell scrapper and the number of cells were counted using a hemocytometer to confirm that an adequate number of cells (> 5.10^6^ cells per ml) were present in the cultures.

### Cell lysis and protein harvest

In the meantime, fresh lysis buffer was prepared using 4.275 ml of buffer mix (7 M urea, 2 M thiourea, 4% CHAPS), 100 µl of 50%, Dithiothreitol (DTT) and 625 µl of a general protease inhibitor cocktail (Fisher Scientific).

At the end of the incubation, the medium was removed and the cells were washed three times with sterile phosphate buffered saline (PBS). Afterwards, the PBS was removed and replaced with 250 µl of the freshly prepared lysis buffer. The lysis buffer was left to act on the cells for 20 min and the cells were harvested using a cell scrapper and placed into a 1.5 ml centrifuge tube.

These lysates were left to incubate in darkness at 4 °C overnight. Afterwards, they were centrifuged at 14,000 RPM at 4 °C for 30 min (cooling centrifuge 5,810 R, Eppendorf) to pellet down cell debris. The supernatants were collected and the protein concentration was measured using the Pierce method (Nanodrop 2000, VWR International). The extracted proteins were stored at 4 °C until analysis, as described below.

### Protein digestion

For each sample, 30 µg of the protein were diluted in 8 M Urea in 50 mM TRIS buffer to a volume of 500 µl before being passed onto a 10 kDa centrifugal filter (Pall) by centrifugation for two times 20 min at 10,000 rcf. Afterwards, the proteins were reduced on filter by addition of 200 mM DTT and incubation for 30 min at 37 °C before being alkylated using 500 mM iodoacetamide (IA) at 37 °C for 30 min. After two washes using 100 µl 50 mM TRIS, digestion was carried out using Trypsin/LysC Mix in a ratio of 1:25 (Protease:Protein) overnight and digested peptides were recovered with three times 50 µl of 50 mM TRIS and acidified using 1 µl of Trifluoroacetic acid (TFA). Peptides were purified using C18 spin columns (Pierce) according to the manufacturer’s instructions and redissolved in 300 µl 0.1% TFA.

### Protein analysis

Three microliters (equivalent to 300 ng of dissolved protein) from each sample were separated on an Ultimate 3,000 RSLC LC system using an Acclaim PepMap C18 column (75 µm inner diameter, 2 µm particle size, and 100 Å pore size) after pre-concentration and desalting on an Acclaim PepMap μ-Precolumn (300 µm inner diameter, 5 µm particle size, and 100 Å pore size).

A 60 min gradient was used to separate the peptides prior to mass spectrometric analysis. The LC was directly coupled to a Q Exactive HF Orbitrap mass spectrometer (Thermo Scientific). Parameters used for MS full scans: m/z ranges: 350–2000; resolution: 60,000; maximum injection time (MIT): 50 ms; automatic gain control: 3.10^6^. MS/MS fragmentation parameter: m/z range: 200–2000; resolution:15,000; intensity threshold: 4.10^3^; normalized collision energy: 28; AGC: 5e^4^; MIT: 50 ms. Only ions with charge state + 2 to + 6 were included.

### Peptide identification and statistical analysis

Identification of the peptides was performed using the Proteome Discoverer Software 2.3.0.523 (Thermo Fisher Scientific) and searching databases of salmonid and *Y. ruckeri* proteins (190408_NCBI_Salmonsalar_tx8030_RefSeq.fasta and 190408_Yersiniaruckeri_tx29486_RefSeq.fasta, respectively). *Salmo salar* was selected, as this database is more complete than that of Chinook salmon (*O. tshawytscha*) while remaining similar enough to allow for a high level of homology.

For the label free quantification, peptide intensities from three technical replicates of each sample were used to calculate differential expression. Therefore the peptide intensities from a label free quantification experiment in Proteome Discoverer 2.3 were filtered as follows: they had to belong to exactly one protein group (only unique peptides) and were not used if they had any modification other than carbamidomethylation. Additional all contaminant proteins (identified in the CRAP database; https://www.thegpm.org/crap/) were excluded.

These peptides were exported to Excel to be further filtered according to missing values. In each group, only two missing values were allowed and all other peptides excluded from the following statistical analysis in R. An exception was made for “on/off” proteins which were peptides with no value in one group but enough values in the other. Abundance values of all peptides of one protein were summed up and proteins with only one peptide were also excluded from the quantification using R Studio.

Data was log-transformed and the median of the technical replicates of each biological replicate was calculated. A t-test was calculated and the resulting *p* values were FDR-adjusted to compensate for multiple testing. The FDR-adjusted *p* values and the fold changes were used for filtering the significant proteins. Proteins that had a FDR adjusted *p* value < 0.05 and at least a twofold change in abundance were considered significantly regulated.

Further analysis was performed by identifying the 20 most upregulated and 20 most down regulated proteins using Uniprot. Furthermore, because there is no protein-interaction network easily available for salmonids, we identified the closest homolog in the genus *Danio* for these 40 proteins, based on homology. Most proteins had a homolog with a high level of similarity (> 70%). For each of these proteins, the interaction network was determined in *Danio* sp. using the STRING database.

## Supplementary information

Supplementary legends (DOCX 11 kb)

Supplementary Table 1 (XLSX 464 kb)

Supplementary Table 2 (XLSX 19 kb)

## Data Availability

The datasets generated during and/or analysed during the current study are available from the corresponding author on reasonable request.
