# Supplementary material for: Effects of Yersinia ruckeri invasion on the proteome of the Chinook salmon cell line CHSE-214
Source: Sci Rep. 2020 Jul 16;10:11840. doi: 10.1038/s41598-020-68903-5 (PMC7366648; doi:10.1038/s41598-020-68903-5)
Supplement: Supplementary file 1 — Supplementary legends (DOCX 11 kb) [file 41598_2020_68903_MOESM1_ESM.docx]

**Supplementary material:**

Supplementary Table 1: List showing the 1614 salmon proteins identified in this study. Highlighted in blue are the differentially expressed proteins.

Supplementary Table 2: List of the 20 most over expressed and 20 most under expressed proteins as well as link to their GO annotation and the STRING analysis of their most similar homolog in *Danio* sp..
